# Do 16 Polycyclic Aromatic Hydrocarbons Represent PAH Air Toxicity?

**DOI:** 10.3390/toxics5030017

**Published:** 2017-08-15

**Authors:** Vera Samburova, Barbara Zielinska, Andrey Khlystov

**Affiliations:** Desert Research Institute, Division of Atmospheric Sciences, 2215 Raggio Parkway, Reno, NV 89512, USA; Barbara.Zielinska@dri.edu

**Keywords:** polycyclic aromatic hydrocarbons, toxic equivalency factor, air samples, gas and particle phase PAHs

## Abstract

Estimation of carcinogenic potency based on analysis of 16 polycyclic aromatic hydrocarbons (PAHs) ranked by U.S. Environmental Protection Agency (EPA) is the most popular approach within scientific and environmental air quality management communities. The majority of PAH monitoring projects have been focused on particle-bound PAHs, ignoring the contribution of gas-phase PAHs to the toxicity of PAH mixtures in air samples. In this study, we analyzed the results of 13 projects in which 88 PAHs in both gas and particle phases were collected from different sources (biomass burning, mining operation, and vehicle emissions), as well as in urban air. The aim was to investigate whether 16 particle-bound U.S. EPA priority PAHs adequately represented health risks of inhalation exposure to atmospheric PAH mixtures. PAH concentrations were converted to benzo(a)pyrene-equivalent (BaPeq) toxicity using the toxic equivalency factor (TEF) approach. TEFs of PAH compounds for which such data is not available were estimated using TEFs of close isomers. Total BaPeq toxicities (∑_88_BaPeq) of gas- and particle-phase PAHs were compared with BaPeq toxicities calculated for the 16 particle-phase EPA PAH (∑_16EPA_BaPeq). The results showed that 16 EPA particle-bound PAHs underrepresented the carcinogenic potency on average by 85.6% relative to the total (gas and particle) BaPeq toxicity of 88 PAHs. Gas-phase PAHs, like methylnaphthalenes, may contribute up to 30% of ∑_88_BaPeq. Accounting for other individual non-EPA PAHs (i.e., benzo(e)pyrene) and gas-phase PAHs (i.e., naphthalene, 1- and 2-methylnaphthalene) will make the risk assessment of PAH-containing air samples significantly more accurate.

## 1. Introduction

Polycyclic aromatic hydrocarbons (PAHs) are widespread organic species in the environment, originating from a variety of sources including wild forest and peat fires [1,2,3], volcano emissions [4,5], and different biological processes [6,7]. For example, biogenic PAHs can be formed due to biosynthesis by microorganisms, phytoplankton, algae, highly developed plants or termite activities [8,9,10]. Atmospheric anthropogenic emissions of PAHs are mainly caused by the combustion of carbon-based fuels [6], such as fossil fuels [11,12,13,14], wood [15,16,17,18], peat [19], agricultural biomass [20,21,22,23], and animal waste [24,25,26]. Anthropogenic emissions of PAHs significantly exceed their natural sources [27,28]. Shen et al. [29] showed that in 2007, global total atmospheric emission of the 16 U.S. EPA priority PAHs (16_EPA_PAHs) was 504 Gg, with biomass fuels combustion, mainly firewood and crop residues, contributing the most (approximately 60.5% of the total global PAH emissions). PAHs are of great environmental concern because of their widespread abundance [1,29,30,31,32,33] combined with high toxicity, mutagenic and/or carcinogenic health effects [34,35]. Carcinogenic effects of PAHs are due to their ability to bind to DNA [36,37,38,39], with many studies showing positive correlation between levels of PAH-DNA adduct formation in different organs and PAH doses [40,41].

There are several routes of PAH exposure, including water, food, tobacco smoke, pharmaceutical products, etc. [35,42,43]. Ambient air is one of the major sources of PAH intake [44,45]. Airborne PAHs are present in both gas and particle phases. Low molecular weight PAHs that have 2–3 aromatic rings in their structure (i.e., naphthalene, methylnaphthalenes, acenaphthylene, fluorene) are mainly present in the gas phase, while heavier PAHs (with four or more aromatic rings, i.e., pyrene, benz(a)anthracene, benzo-fluoranthenes) are usually associated with atmospheric particulate matter (PM) [46,47,48,49]. The U.S. EPA set the regulation for 16 PAHs [50], which were selected based on knowledge of their relative toxicity, abundance, chance of exposure, and levels in the environmental samples [35]. TEFs have been only determined for 17 reasonably-well studied PAHs [51]—the 16 EPA priority PAHs (Table S1) and 2-methylnaphthalene. However, the number of PAHs present in the environment is significantly larger and very little is known about their carcinogenic properties [35]. For this reason, most of air pollution studies have focused on sources, emission factors, and toxic effects of these 16-17 PAHs [2,23,34,52,53,54]. Since nine out of the 16 EPA PAHs are mostly present in the particle phase, research has been focused on analysis of particle-bound PAHs, largely ignoring gas-phase PAHs. Yet, gas-phase PAHs may have a strong carcinogenic health impact [35,42]. For example, several toxicological studies reported a link between naphthalene exposure and a number of adverse health outcomes such as nasal cancer [55,56,57,58]. It needs to be emphasized, that only a very little fraction of this compound (a few %) is found in the particle phase and most of naphthalene mass is usually present in the gas phase [48,59,60].

In order to estimate health effects of different pollutants, the toxicity equivalency factor (TEF) methodology was developed by the U.S. Environmental Protection Agency (EPA) [61] and adapted for PAH compounds [51]. TEF represents the toxicity of an individual PAH compound relative to the reference chemical—benzo(a)pyrene. Benzo(a)pyrene is the most studied PAH for carcinogenic properties [62] and, for this reason, it was selected as the reference compound. To calculate the toxicity potential of a specific PAH, its concentration is multiplied by the determined TEF value. The total potential carcinogenic potency of PAH mixtures in air samples is determined by summing up concentrations of individual PAHs, which are multiplied by the determined TEFs of individual PAHs [51]. The International Agency for Research on Cancer (IARC) classifies PAHs by their toxic potencies as probable (2A) and possible (2B) carcinogens [35] and the following PAHs have been highlighted: benzo(a)pyrene, dibenz(a,h)anthracene, benz(a)anthracene, chrysene, benzo(b)fluoranthene, benzo(k)-fluoranthene, benzo(ghi)perylene, and indeno(1,2,3-c,d)pyrene.

Only a few studies have performed quantitative analyses on more than the 16 airborne PAHs for both gas and particle phases. Our group performed measurements of over 70 gas and particle phase PAHs in different vehicle emissions [11,63,64], biomass-burning [65,66], and meat cooking samples [67]. Kameda et al. [68] collected filters and polyurethane foam (PUFs) cartridges to analyze 22 gas and particle-phase atmospheric PAHs (16 EPA priority PAHs and additional high risk PAHs recommended by WHO/IPCS [69]) to evaluate PAH toxicity and to study deposition of these species in different regions of the human respiratory tract. The 16 EPA priority PAHs were analyzed by Black et al. [70] and Cereceda-Balic et al. [71] to characterize the distribution of these compounds between gas and particle phases in biomass-burning emissions. Analysis of gas-particle distribution of urban and suburban PAHs was presented by several groups [14,46,48,72]. In Vasilakos [72] and Gregoris [47] studies, for example, Teflon-impregnated glass fiber (TIGF) filters and PUFs were used to collect particle and gas-phase species, but concentration levels of only the 16 specified PAHs were measured.

Most health risk assessment studies have also been focused on the analysis of only the 16 EPA priority PAHs, with PM-bound PAHs being analyzed most extensively. Muendo et al. [73] presented measurements of 25 PAHs; however only filter samples were collected and characterized for particulate PAHs. Delgado-Saborit et al. [74] estimated the carcinogenic potential of 16 particle-phase PAHs in different outdoor and indoor environments. Sources and cancer risk assessments of 26 PM_2.5_-bound PAHs were analyzed by Bandowe et al. [75] in the air of a mega city in China. Allen et al. [76] measured a total of 41 PAHs at rural and urban Massachusetts areas to study the distribution of these PAHs between particles of different sizes. However, the scope of these studies with extended lists of analyzed PAHs has been limited only to the analysis of particle phase.

Many studies [35] have used the 16 U.S. EPA PAHs due to their recognized toxicity by the scientific community in the late 1960s and early 70s. Analytical capabilities and standard materials available at that time imposed some limitations on the selection of PAHs and thus further health risk assessment. At present, a larger number of PAH compounds can be analyzed simultaneously. That opens a discussion for whether other PAH compounds should be recognized as priority PAHs and studied for their toxic properties.

This paper presents an analysis of results from a unique set of 13 projects in which 88 PAHs were measured in both particle and gas phases. PAH samples were collected from different types of sources (biomass burning, vehicle emissions, meat cooking, mining operations and urban air). The goal of this study was to compare TEF-based carcinogenic potency of the 16 U.S. EPA PAHs with the total TEF-based carcinogenic potency of the measured 88 PAHs in both phases and to determine whether measurements of only the 16 U.S. EPA particle-bound PAHs adequately represent the total potential toxicity of PAHs originating from different sources. Using the TEF approach, the relative importance of gaseous and particulate PAHs, as well as the most prominent contributors to the total PAH toxicity in different sources were determined.

## 2. Experimental

### 2.1. Materials and Reagents

Certified PAH standards were purchased from Sigma-Aldrich (St. Louis, MO, USA), AccuStandard (New Haven, CT, USA), and Cambridge Isotope Laboratories, Inc. (Andover, MA, USA). Acetonitrile, acetone, methanol, and dichloromethane (High-Performance Liquid Chromatography grade) were obtained from Fisher Scientific (Fair Lawn, NJ, USA). TIGF filters (Fiber Film T60A20, PALL Life Sciences, Ann Arbor, MI, USA) and XAD-4 resin (Amberlite^®^ XAD4, St. Louis, MO, USA) were used as sampling media. Prior to sampling, XAD-4 resin was cleaned using an accelerated solvent extractor (ASE, DIONEX, ASE-300, Salt Lake City, UT, USA) with methanol followed by dichloromethane for 15 min each at 85 °C and 1500 psi pressure. Clean XAD-4 resin was dried in a vacuum oven at room temperature overnight. Glass (Tisch Environmental, Inc., Village of Cleves, OH, USA) and stainless-steel Teflon-coated cartridges (homemade DRI’s cartridges) were packed with 20–200 g of XAD-4 resin depending on the project (Table 1) and expected concentration levels of PAHs. Every 10th blank XAD-4 cartridge and TIGF filter were extracted on ASE and analyzed with gas chromatography mass spectrometry (GC-MS) to determine their purity. Ultra-high purity (UPH) grade nitrogen was purchased from Airgas (Sparks, NV, USA).

### 2.2. Sampling

Gas- and particle-phase PAH concentrations were measured during several projects aimed at the characterization of: urban ambient air (A-1, A-2), biomass burning (B-1), meat cooking (M-1), engine exhaust (E1–E5), traffic emissions in a road tunnel (T-1, T-2), and mining operations (Mi-1, Mi-2) (Table 1). In total, 325 filter and XAD samples (650 individual samples) were collected, extracted, and analyzed. Detailed descriptions of sampling campaigns can be found in previously published papers and reports (Table 1). Briefly, particulate matter (PM) was collected using medium and high-volume samplers at different flow rates depending on the project (Table 1). TIGF filters (Fiber Film T60A20, PALL Life Sciences, Ann Arbor, MI, USA) were used to collect PM for PAH analysis. Gas-phase semi-volatile PAHs were collected with XAD-4 resin cartridges that were placed downstream of the filters.

After sampling, each filter and XAD cartridge was packed into pre-cleaned aluminum foil, sealed in secure static-sensitive double-track zipper bags (Uline, Pleasant Prairie, WI, USA), and transported in a cooler with blue ice to DRI, where it was stored at −20 °C until extraction and analysis. A detailed description of the extraction and GC-MS analysis are described elsewhere [13,79].

### 2.3. Calculation of BaPeq Toxicity

To estimate the carcinogenic potency of analyzed PAHs, TEF values proposed by Nisbet and LaGoy [51] were applied. TEFs are widely used to calculate toxicities of PAH mixtures based on the toxicity of a specific PAH compound relative to the toxicity of the reference compound—benzo(a)pyrene. The potential cancer risk associated with exposure to PAH mixtures is calculated by summing up the concentrations of individual PAH compounds, multiplied by their TEF values (Table S1). TEF values were not available for all 88 PAHs measured in this study. TEFs have been determined for only 17 PAHs [51], including the 16 EPA priority PAHs [acenaphthylene, acenaphthene, fluorine, phenanthrene, anthracene, fluoranthene, pyrene, benz(a)anthracene, chrysene, benzo(b)fluoranthene, benzo(k)fluoranthene, benzo(a)pyrene, dibenz(a,h)anthracene, benzo(ghi)perylene, indeno(1,2,3-cd)pyrene), and 2-methylnaphthalene (Table S1)]. TEFs for the remaining 71 PAHs were estimated based on the similarity of a compound to one of the 17 PAHs whose TEFs are known. Compounds were considered similar if they are close isomers, have the same number of aromatic rings, GC-MS response factors, and similar molecular structure. For example, for 1-methylnaphthalene, the TEF of 2-methylnaphthalene (0.001) [51] was used since these compounds are isomers and are structurally similar. The same TEF (0.001) was applied for dimethyl- and trimethylnaphthalenes, because GC-MS response factors of these PAHs are similar to methylnaphthalene and because chemical structures of these compounds differ only by the presence of one or two methyl (-CH_3_) groups. The TEF proposed for anthracene (0.01) was used to calculate the BaPeq concentrations for methylanthracenes and different benz(a)anthracenes like 7-methylbenz(a)anthracene and 7,12-dimethylbenz(a)anthracene. In the case of fluoranthenes [1 + 3-methylfluoranthene, 4-methylfluoranthene, benzo(ghi)fluoranthene, etc.], TEF of fluoranthene (0.001) was applied. TEFs of four isomers of dibenzopyrene [dibenzo(a,e)pyrene (TEF: 1), dibenzo(a,l)pyrene (TEF: 10), dibenzo(a,i)pyrene (TEF: 10), dibenzo(a,h)pyrene (TEF: 10)] were assigned using values provided by Office of Environmental Health Hazard Assessment (OEHHA) report [80]. It has to be emphasized that TEFs assigned based on analytical criteria described above may not adequately reflect the real toxicological properties of non-EPA PAHs. Since to our knowledge, there are no toxicological studies on these compounds, we used this approach to estimate the relative importance of the non-EPA PAHs and selected those that deserved a more thorough toxicological characterization. It should be also noted that 75% of the assigned TEFs (Table S2) had the lowest TEF value (0.001), which makes overestimation of their toxicity less likely. Table S2 summarizes TEFs that were assigned to the 88 PAHs measured in this study. BaPeq concentrations of PAH mixtures in different samples were then calculated using the following formula:

(1) $$\sum 88BaPeq=\overset{i=88}{\underset{i=1}{\sum }}Ci\times TEFi$$

where *Ci*—concentration of PAH compound *i* (in ng m^3^); *TEFi*—assigned toxic equivalency factor (Table S2).

Total BaPeq concentrations of 16 EPA priority PAHs (∑_16_BaPeq) were calculated using the same formula, but this was applied only to the 16 EPA PAHs [51].

## 3. Results and Discussion

Measured PAH concentrations varied significantly from project to project, because of the differences in the nature of each sampling project (see Experimental Section). For example, the concentration of gas-phase naphthalene measured during controlled combustion of five different fuels [78] was 2637 ± 929 ng m^−3^ on average, while its concentration in ambient urban air in downtown Reno (NV, USA) was more than 10 times lower (167 ± 71 ng m^−3^). To estimate the carcinogenic potency of PAH-containing samples (Table 1), the sum of BaPeq concentrations of 88 (∑_88_BaPeq) and 16 (∑_16_BaPeq) gas- and particle-phase PAHs, respectively, were calculated for each project and summarized in Table S3. As expected, ∑BaPeq values vary significantly from project to project because of the differences in PAH concentrations observed during each project (Table 1). For example, the mean ∑_88_BaPeq value for particle-phase PAHs during the biomass-burning project was 41.3 ± 22.1 ng m^−3^, which was significantly higher than that of the measured urban PM (project A-1), where the ∑_88_BaPeq was 0.341 ± 0.242 ng m^−3^.

To compare the total BaPeq toxicity of the 16 EPA particle-phase PAHs with that of 88 PAHs (∑_88_BaPeq), as well as with BaPeq toxicities that include gas-phase PAHs, the following ∑BaPeq ratios were calculated for each sample collected during the 13 projects considered here (Table S4):

(2) $$BaPeq{\left(\%\right)}_{I}=\frac{\mathrm{PM}\sum_{16}\mathrm{BaPeq}}{\mathrm{PM}\sum_{88}\mathrm{BaPeq}}\times 100\%$$

(3) $$BaPeq{\left(\%\right)}_{II}=\frac{\mathrm{PM}\sum_{16}\mathrm{BaPeq}}{\left(\mathrm{PM}+\mathrm{GAS}\right)\sum_{16}\mathrm{BaPeq}}\times 100\%$$

(4) $$BaPeq\;{\left(\%\right)}_{III}=\frac{\mathrm{PM}\sum_{16}\mathrm{BaPeq}}{\left(\mathrm{PM}+\mathrm{GAS}\right)\sum_{88}\mathrm{BaPeq}}\times 100\%$$

The above ratios helped us to assess whether the 16 EPA particle-bound PAHs were sufficient for evaluation of the total PAH toxicity of aerosols originating from different sources. Ratios close to 1 would indicate that the 16 EPA PAHs adequately represented the total toxicity, while lower ratios will provide an estimate of how much the PAH toxicity is underestimated by the traditional 16-PAH method. Summary statistics of these ratios for each project are given in Table S4. For simplicity, only the mean values of the ∑BaPeq ratios will be discussed in the text below.

**Figure 1:**
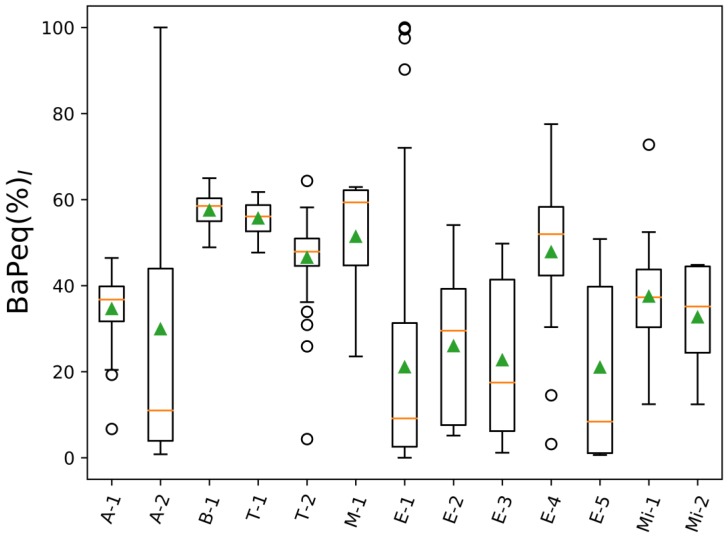
BaPeq ratios of 16 U.S Environmental Protection Agency (EPA) particle-phase PAHs to 88 particle-phase PAHs (BaP_eq_(%)_I_ = PM∑_16EPA_BaP_eq_/PM∑_88_BaP_eq_); orange lines—mean values of BaP_eq_(%)_I_; green triangles—median values of BaP_eq_(%)_I_.

Figure 1 presents a graphical summary of BaPeq(%)_I_ ratios [Equation (2)]. BaPeq(%)_I_ that considered only particle-phase PAHs did not exceed 60% in any of the analyzed projects. The particle-bound 16 EPA priority PAHs were found to be responsible, on average, only for 21–58% of the PM PAH toxicity (Table S4). The lowest project-average BaPeq(%)_I_ (~21%) was observed for heavy-duty vehicle engine emissions (project E-5, Table S4). It should be noted that within each project there were BaPeq(%)_I_ observations that were significantly lower than the project-average values. These results indicated that PAHs not included in the EPA priority list contributed more to the PM toxicity than the 16 EPA PAHs, especially in the case of heavy-duty vehicle emissions (E-projects, Table 1 and Table S4). Analysis of BaPeq values for individual 88 PAHs showed the dominance of dibenzo(a,i)pyrene (25%), benzo(e)pyrene (24%), benzo(a)pyrene*_EPA_* (14%), benz(a)anthracene*_EPA_* (11%), dibenzo(a,h)pyrene (9%), and benzo(b)fluoranthene*_EPA_* (5%) in the total PM∑_88_BaPeq toxicity of heavy-duty vehicle emissions (Figure S1, project E-5). As can be seen, only three out of six listed top PAHs were EPA priority compounds, which accounted for only 31% of PM∑_88_BaPeq levels. In the case of biomass-burning samples (project B-1), the EPA priority PAHs contributed more to the total PM PAH toxicity (∑_88_BaPeq) than to the heavy-duty engine emissions (E-1), on average making up to 57.5% of the total PM∑_88_BaPeq. The dominant PAHs in biomass-burning samples were benzo(a)pyrene*_EPA_* (37%), benzo(e)pyrene (30%), benz(a)anthracene*_EPA_* (6%), benzo(b)fluoranthene*_EPA_* (5%), dibenzo(ah)anthracene*_EPA_* (4%), benzo(j)fluoranthene (3%). Overall, non-EPA PAHs were responsible for 42.5% of the PM∑_88_BaPeq (Figure S1, project B-1), which was still a significant contribution to the PAH toxicity level. Delgado-Saborit et al. [74] reported that the main contributor to the carcinogenic potency of PM PAH mixtures in different indoor and outdoor environments is benzo(a)pyrene, which was in agreement with our results. However, in their study, only 16 EPA PAHs were measured, and thus the carcinogenic potency of non-EPA PAHs has been overlooked.

**Figure 2:**
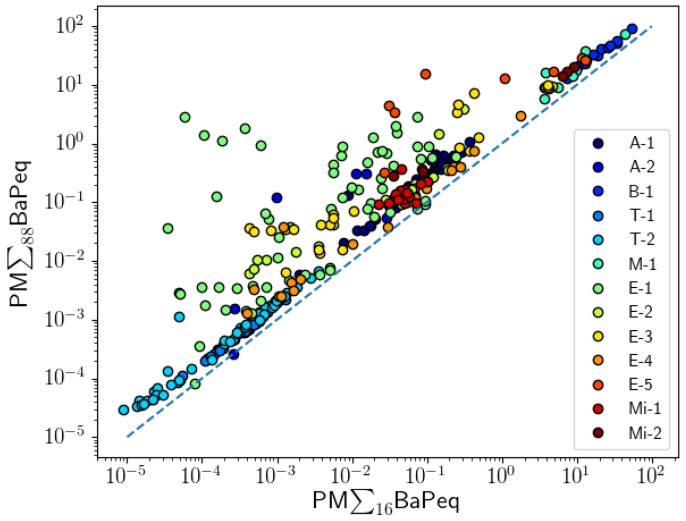
Correlation between BaPeq of 88 PAHs in particle-phase (PM) and 16 EPA particle-phase PAHs analyzed for 13 projects (Table 1).

A comparison between PM BaPeq values of the 16 EPA priority PAHs and the 88 analyzed PM PAHs showed positive liner correlations for all projects (Figure 2, Table 2). The correlations were calculated using Spearman’s approach. A strong linear relationship was observed for most of the projects: both tunnel studies (T-1: r = 0.981; T-2: r = 0.987), PM samples collected at the LAX airport (A-1: r = 0.917), during controlled biomass combustion experiments (B-1, r = 0.991), one project on mining pollutants (Mi-2: r = 0.943), and analysis of meat cooking emissions (M-1: r = 0.893). Good correlations between BaPeq of the 16 EPA PAHs and those of the 88 PAHs were observed for PM emissions from vehicle engines, except for the E-1 project where a poor correlation was obtained (r = 0.507). Poor correlations were also observed for one mining project (Mi-1: r = 0.450) and for the filter samples collected in the Reno city area (A-2, r = 0.657). The lack of a correlation (PM∑_16_BaPeq and PM∑_88_BaPeq) shows that, in general, the total ∑BaPeq toxicity cannot be reliably extrapolated based on measurements of the 16 EPA priority PAHs.

As was mentioned above, many ambient PAH studies have been focused on characterization of particle-phase PAHs, while gas-phase PAH species have been underrepresented in air quality projects so far. It has been emphasized in the literature [35,81] that high molecular weight PAH compounds (with four or more aromatic rings) are more toxic, and thus analysis of PM, especially PM_2.5_, is more important than gas phase measurements. At the same time, light PAHs with 2–3 aromatic rings, such as naphthalene and naphthalene derivatives, are mainly present in the gas phase. Measurements of only PM PAHs, thus could underestimate the total PAH toxicity of air samples [60]. The contribution of gas phase PAHs to the total PAH carcinogenic potency was evaluated using two equations: the BaPeq(%)_II_ ratio [Equation (3)] and the BaPeq(%)_III_ ratio [Equation (4)].

**Figure 3:**
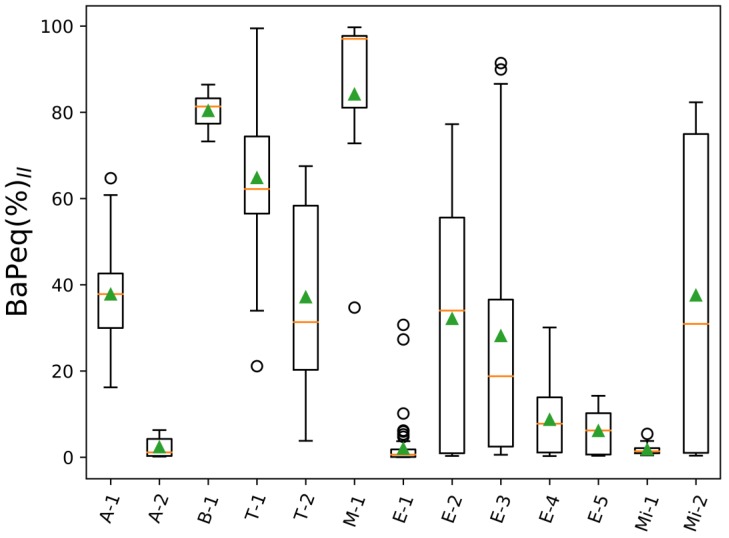
BaPeq ratios of 16 EPA priority particle-phase PAHs to 16 EPA priority PAHs in both gas and particle phases (BaP_eq_(%)_II_ = PM∑_16EPA_BaP_eq_/(PM + Gas)∑_16EPA_BaP_eq_); orange lines—mean values of BaP_eq_(%)_II_; green triangles—median values of BaP_eq_(%)_II_.

Equation (3) represents the contribution of the particle phase of the 16 EPA priority PAHs to their total (gas + particles) toxicity, (PM + Gas)∑_16_BaPeq. BaPeq(%)_II_ ratios for all analyzed projects are summarized in Figure 3 and Table S4. The ratios varied significantly from project to project, and ranged between 1.8% and 84%. A dominant contribution of the 16 gas-phase EPA priority PAHs to (PM + Gas)∑_16_BaPeq was observed for A-2, E-1, E-4, E-5, and Mi-1 projects, where the 16 EPA particle-bound PAHs were responsible only for 2.3, 2.0, 8.7, 6.1, and 1.8%, respectively. The contribution of the gas-phase PAHs to the total toxicity of the 16 EPA was also larger than that of the particle-phase 16 PAHs for most of the other projects (A-1, T-2, E-2, E-3, Mi-2)—the particle-phase 16 PAHs contributed, on average, between 28% and 38%. Only for three projects (B-1, T-1, and M-1), the 16 particle-bound EPA PAHs had a significant contribution to the (PM + Gas)∑_16_BaPeq toxicity—above 50%. Moreover, we also found that within one project, the distribution of PAHs between gas and particle phases and, therefore, their PM BaPeq contribution, varies significantly. Our study of traffic emissions at the Fort McHenry tunnel (Baltimore, MD, USA) showed that the contribution of the particle-phase EPA PAHs to the total 16 PAH toxicity (or ∑_16_BaPeq) is larger in winter (T-1, BaPeq(%)_II_ = 64.8%) than in summer (T-2, BaPeq(%)_II_ = 37.1%). During this study, summer temperatures were on average 29 °C, ranging from 25 °C to 31 °C, while in winter, temperatures were on average 0.2 °C, ranging from −9.1 °C to 13.7 °C. Gas-particle partitioning of semi-volatile compounds shifts to the gas phase at higher temperature [48,82,83], which explains the stronger contribution of gas-phase PAHs during summer months of this project. It should be also noted that the gas-particle partitioning of PAHs and other semi-volatile compounds depends not only on ambient temperature, but also on the concentration of particle-phase organics [83]. Thus, dilution of emitted PAHs and mixing with organic aerosols from other sources could change gas-particle partitioning of semi-volatile PAHs. Measurements of only particle-phase PAHs represent particle-phase toxicity only at the conditions of the measurements and cannot be used to estimate toxicity at other conditions.

**Figure 4:**
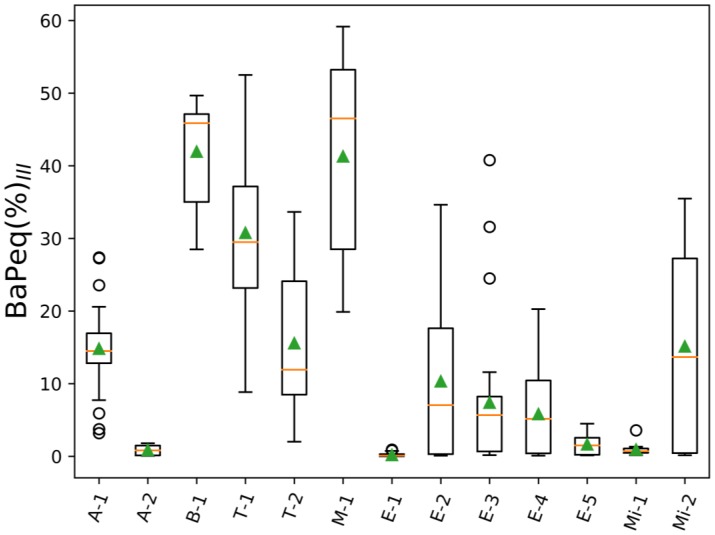
BaPeq ratios of 16 EPA particle-phase PAHs to 88 PAHs for both gas and particle phases: BaP_eq_(%)_III_ = PM∑_16EPA_BaP_eq_/(PM + Gas)∑_88_BaP_eq_; orange lines—mean values of BaP_eq_(%)_III_; green triangles—median values of BaP_eq_(%)_III_.

**Figure 5:**
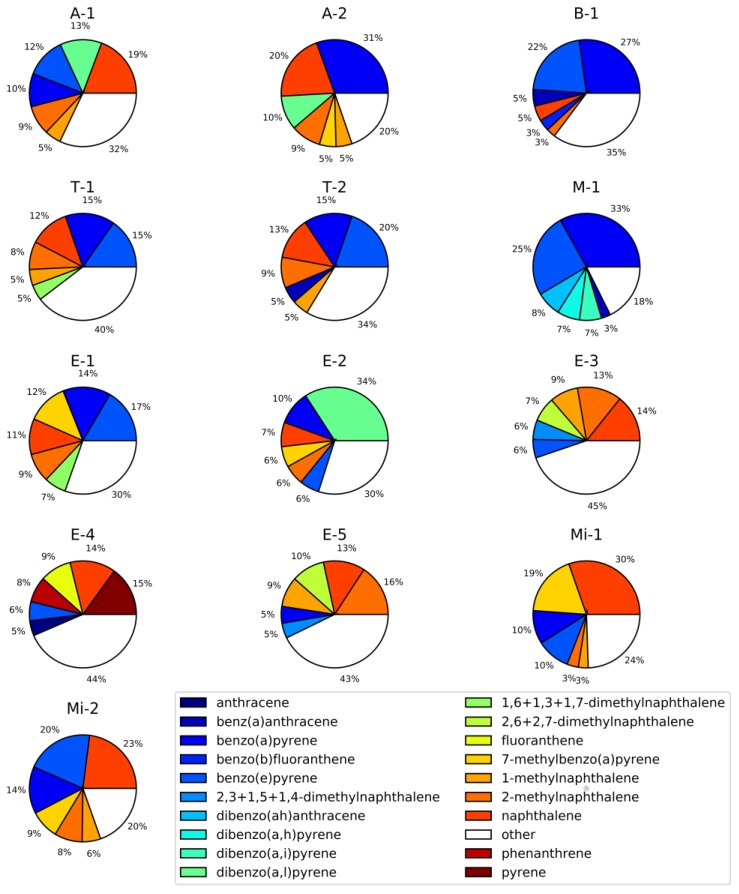
Top six PAHs that have highest BaPeq concentration levels in both gas and particle phases.

To illustrate how much the PAH carcinogenic potency of analyzed air samples would change if 88 gas- and particle-phase PAHs are taken into account instead of only 16 particle phase EPA priority PAHs, BaPeq(%)_III_ ratios [Equation (4)] were calculated (Table S4, Figure 4). BaPeq(%)_III_ ratios for six projects (B-1, E-1, E-3, E-4, E-5, Mi-1) were below 10% (range: 0.2–7.4%); for four projects (A-1 T-2, E-2, Mi-2) these ratios were not higher than 20% (A-1: 14.8%; T-2: 15.6%; E-2: 10.3%; Mi-2: 15.1%); and for the remaining three projects BaPeq(%)_III_ values were 41.9% (B-1), 30.8% (T-1), and 41.3% (M-1). As would be expected, all BaPeq(%)_III_ ratios were significantly lower (2–10 times) than BaPeq(%)_II_, since the contribution of all 88 PAHs and the gas phase were taken into account. Therefore, assuming that our assignment of TEF values to the non-EPA PAHs adequately represents their benzo(a)pyrene equivalent toxicity, the 16 EPA PAHs significantly underestimated the carcinogenic potency of PAH mixtures in all of the scenarios considered in this study. Moreover, several toxicological studies emphasized that PAHs in mixtures can have additive or synergistic health effects [84,85] and thus, risk assessment based on limited number PAH compounds most likely underestimates the total carcinogenic potency of complex PAH mixtures. Figure 5 shows the top six contributors to the total BaPeq toxicity ([PM + Gas]∑_88_BaPeq) for each individual project. In the majority of the projects, the following compounds substantially contributed to the total BaPeq toxicity: benzo(a)pyrene (16.8 ± 9.2%), naphthalene (15.1 ± 7.1%), benzo(e)pyrene (14.4 ± 7.0%), 2-methylnaphthalene (8.6 ± 3.8%), and 1-methylnaphthalene (5.7 ± 2.1%). It has to be noted that three out of five top listed PAHs were not the EPA priority compounds (1-methylnaphthalene, 2-methylnaphthalene, and benzo(e)pyrene). Moreover, 1- and 2-methylnaphthalenes are low molecular weight PAHs, which are usually found in the gas phase [47,48,72,82]. For some of the projects different PAHs contributed significantly to the overall and gas-phase BePeq toxicity. For example, 2,4,5-trimethylnaphthalene was the second top contributor in the gas phase biomass-burning emissions (project B-1, 9.9%), while for mining samples (Mi-1), 7-methylbenzo(a)pyrene was the second top gas phase PAH, contributing 18.9% to the ∑_88_BePeq toxicity (Figure S2).

**Figure 6:**
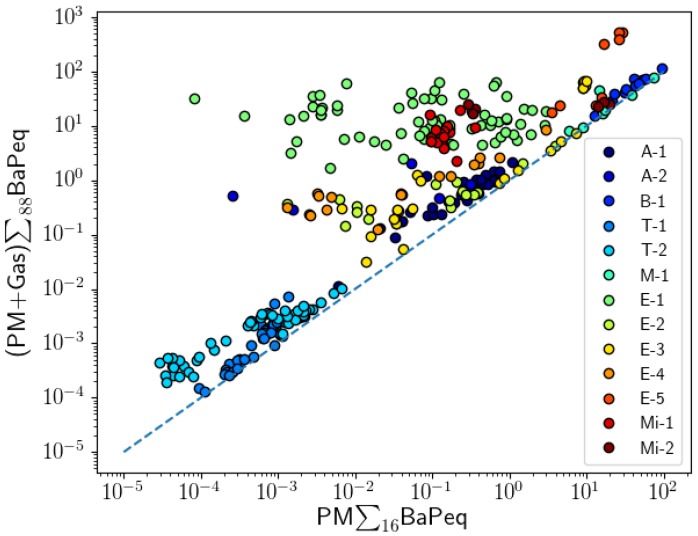
BaPeq correlation between 16 EPA particle-phase PAHs and 88 PAHs in both gas and particle phases.

It is clear that the “PM-only analysis” approach could significantly underestimate the carcinogenic potency of PAH mixtures. Similar conclusions have been presented by Ramirez et al. [60]. In their work, 18 gas and particle-phase PAHs have been analyzed in air samples collected near industrial cities, and the contribution of gas-phase PAHs to the total BaPeq was found to be significant (34–86%). The 16 EPA PAHs could still be used for toxicity predictions if their BaPeq toxicity correlates well with the BaPeq toxicity of all PAH compounds in the mixture. Correlation coefficients of PM∑_16_BaPeq with (PM + Gas)∑_88_BaPeq are presented in Figure 6 and Table 2. Significantly positive correlations (r > 0.8) were observed for eight projects (A-1, A-2, B-1, T-1, T-2, M-1, E-3, E-4, E-5), suggesting that the total carcinogenic potency observed in these projects could be estimated using measurements of only the 16 EPA particle-phase PAHs. However, poor correlations were observed among similar types of PAH sources: E-1 (r = 0.054) vs. E-5 (r = 0.917). This shows that applying a correction factor to PM∑_16_BaPeq to estimate PM∑_16_BaPeq may not be applicable in all conditions and could introduce a large uncertainty in toxicity estimates. 

## 4. Conclusions

In the present study, the data of 13 different projects (650 filters and XAD cartridges, total) on the analysis of 88 gas and particle-phase PAHs were used to calculate the total BaPeq toxicity by applying a widely used TEF approach. Obtained BaPeq values were compared with those calculated for 16 EPA priority particle-phase PAHs, and we found that 16 particle-bound EPA PAHs were responsible for only 14.4% on average for the range in all projects (0.2–41.9%) and the health risk of analyzed samples was likely to have been hugely underestimated. Although there was a significant linear relationship between PM∑16BaPeq and PM∑88BaPeq as well as between PM∑16BaPeq and (PM+Gas)∑88BaPeq for a majority of the analyzed projects, the potential carcinogenic toxicity based on BaPeq concentrations for only 16 EPA particle-phase PAHs must be interpreted carefully, especially considering possible synergistic effects of PAH mixtures. Our findings on the analysis of BaPeq values for 88 gas and particle phase individual PAH compounds showed that the contribution of non-EPA PAHs such as 1- and 2-methylnaphthlalenes, benzo(e)pyrene, dibenzo(a,i)pyrene, dibenzo(a,h)pyrene, is substantial to carcinogenic potency of analyzed air samples. 

Our results stress the need for data on TEF values for additional PAHs, and the incorporation of these compounds into the health risk assessment. Measurements of gas-phase PAHs are also recommended, because of the strong contribution of the gas phase to the total BaPeq toxicity of atmospheric PAHs. 

## Figures and Tables

**Table 1:** List of projects and number of collected filter and XAD samples that were analyzed for PAHs in particle and gas phases, respectively.

Project Abbreviation and Reference	Sample Source	Description	Number of Samples	
			Filters	XAD
**A-1 [77]**	Ambient urban air	LAX airport (CA)	44	44
**A-2**	Ambient urban air	Reno city (NV)	6	6
**B-1 [78]**	Controlled biomass burning	DRI combustion chamber (NV)	11	11
**T-1**	Tunnel/car emissions	Fort McHenry tunnel (MD), winter 2015	46	46
**T-2**	Tunnel/car emissions	Fort McHenry tunnel (MD), summer 2015	50	50
**M-1**	Meat cooking	-	7	7
**E-1**	Engine emissions	Heavy and light duty engines	71	71
**E-2**	Engine emissions	Engine emissions (Cummins)	16	16
**E-3**	Engine emissions	Engine emissions (Cummins)	26	26
**E-4**	Engine emissions	Engine emissions (Cummins)	17	17
**E-5**	Engine emissions	Emissions from heavy heavy-duty diesel engines (model year 2007)	9	9
**Mi-1**	Mining	Mining operation facility	16	16
**Mi-2**	Mining	Mining operation facility	6	6

**Table 2:** BaPeq correlations of 16 EPA priority particle-phase PAHs (PM∑_16_BaPeq) with a BaPeq of 88 particle-phase PAHs (PM∑88BaPeq) and BaPeq of both particle- and gas-phase PAHs. The calculations were performed using Spearman’s correlation.

Project	PM∑16BaPeq/PM∑88BaPeq		PM∑16BaPeq/(PM + GAS)∑88BaPeq	
	r_Value	*p*_Value	r_Value	*p*_Value
A-1	0.917	0.0000	0.841	0.0000
A-2	0.657	0.1562	0.829	0.0416
B-1	0.991	0.0000	0.936	0.0000
T-1	0.981	0.0000	0.848	0.0000
T-2	0.987	0.0000	0.919	0.0000
M-1	0.893	0.0068	0.857	0.0137
E-1	0.507	0.0000	0.054	0.6580
E-2	0.982	0.0000	0.688	0.0032
E-3	0.888	0.0000	0.857	0.0000
E-4	0.941	0.0000	0.838	0.0000
E-5	0.917	0.0005	0.917	0.0005
Mi-1	0.450	0.0803	−0.297	0.2639
Mi-2	0.943	0.0048	0.543	0.2657

## Supplementary Materials

The following are available online at www.mdpi.com/2305-6304/5/3/17/s1, Table S1: EPA priority PAHs and TEF coefficients, Table S2: TEFs assigned to 88 gas and particle phase PAHs analyzed for 9 different projects, Table S3: BaPeq calculated for samples collected for different projects, Table S4: Ratios of BaPeq calculated for samples collected for different projects, Figure S1: Top six PAHs that have highest BaPeq concentrations in particle-phase samples, Figure S2: Top six PAHs that have highest BaPeq concentrations in gas-phase samples.
