# Supplementary material for: Do 16 Polycyclic Aromatic Hydrocarbons Represent PAH Air Toxicity?
Source: Toxics. 2017 Aug 15;5(3):17. doi: 10.3390/toxics5030017 (PMC5634701; doi:10.3390/toxics5030017)
Supplement: Supplementary file 1 [file toxics-05-00017-s001.pdf]

Supplementary Materials: Do 16 Polycyclic Aromatic Hydrocarbons Represent PAH air toxicity?

Vera Samburova, Barbara Zielinska and Andrey Khlystov

Table S1. EPA priority PAHs and TEF coefficients.

Name	EPA priority PAHs	TEF (Nisbet et al.)	# of aromatic rings
Naphthalene	x	0.001	2
Acenaphthylene	x	0.001	2
Acenaphthene	x	0.001	2
Fluorene	x	0.001	2
Phenanthrene	x	0.001	3
Anthracene	x	0.01	3
Fluoranthene	x	0.001	3
Pyrene	x	0.001	4
Benz[*a*]anthracene	x	0.1	4
Chrysene	x	0.01	4
Benzo[*b*]fluoranthene	x	0.1	4
Benzo[*k*]fluoranthene	x	0.1	4
Benzo[*a*]pyrene	x	1	5
Dibenz[*a,h*]anthracene	x	5	5
Benzo[*ghi*]perylene	x	0.01	6
Indeno[*1,2,3-cd*]pyrene	x	0.1	5
2-Methylnaphthalene		0.001	2

Table S2. TEFs assigned to 88 gas and particle phase PAHs analyzed for 9 different projects.

PAH #	Compound	TEF	TEFs to isomers are assigned & literature	Used standard
1	naphthalene	0.001	0.001	naphthalene
2	2-methylnaphthalene	0.001	0.001	2-methylnaphthalene
3	1-methylnaphthalene		0.001	2-methylnaphthalene
4	2,6+2,7-dimethylnaphthalene		0.001	2-methylnaphthalene
5	1,3+1,6+1,7dimethylnaphth		0.001	2-methylnaphthalene
6	1,4+1,5+2,3-dimethylnaphth		0.001	2-methylnaphthalene
7	Acenaphthylene	0.001	0.001	
8	1,2-dimethylnaphthalene		0.001	2-methylnaphthalene
9	1,8-dimethylnaphthalene		0.001	2-methylnaphthalene
10	Acenaphthene	0.001	0.001	
11	1+2ethylnaphthalene		0.001	2-methylnaphthalene
12	1-ethyl-2-methylnaphthalene		0.001	2-methylnaphthalene
13	2,3,5+I-trimethylnaphthalene		0.001	2-methylnaphthalene
14	B-trimethylnaphthalene		0.001	2-methylnaphthalene
15	A-trimethylnaphthalene		0.001	2-methylnaphthalene
16	C-trimethylnaphthalene		0.001	2-methylnaphthalene

Table S2. *Cont.*

PAH #	Compound	TEF	TEFs to isomers are assigned & literature	Used standard
17	2-ethyl-1-methylnaphthalene		0.001	2-methylnaphthalene
18	E-trimethylnaphthalene		0.001	2-methylnaphthalene
19	2,4,5-trimethylnaphthalene		0.001	2-methylnaphthalene
20	F-trimethylnaphthalene		0.001	2-methylnaphthalene
21	Fluorene	0.001	0.001	
22	1,4,5-trimethylnaphthalene		0.001	2-methylnaphthalene
23	J-trimethylnaphthalene		0.001	2-methylnaphthalene
24	A-Methylfluorene		0.001	Fluorene
25	B-Methylfluorene		0.001	Fluorene
26	1-Methylfluorene		0.001	Fluorene
27	Phenanthrene	0.001	0.001	
28	3-methylphenanthrene		0.001	Phenanthrene
29	2-methylphenanthrene		0.001	Phenanthrene
30	4,5-methylenephenanthrene		0.001	Phenanthrene
31	9-methylphenanthrene		0.001	Phenanthrene
32	1-methylphenanthrene		0.001	Phenanthrene
33	A-dimethylphenanthrene		0.001	Phenanthrene
34	B-dimethylphenanthrene		0.001	Phenanthrene
35	1,7-dimethylphenanthrene		0.001	Phenanthrene
36	3,6-dimethylphenanthrene		0.001	Phenanthrene
37	D-dimethylphenanthrene		0.001	Phenanthrene
38	E-dimethylphenanthrene		0.001	Phenanthrene
39	C-dimethylphenanthrene		0.001	Phenanthrene
40	Fluoranthene	0.001	0.001	
41	Pyrene	0.001	0.001	
42	Retene		0.001	Fluorene
43	benzo(a)fluorene		0.001	Fluorene
44	benzo(b)fluorene		0.001	Fluorene
45	B-MePy/MeFl		0.001	Fluoranthene
46	1+3-methylfluoranthene		0.001	Fluoranthene
47	4-methylpyrene		0.001	Fluoranthene
48	C-MePy/MeFl		0.001	Fluoranthene
49	D-MePy/MeFl		0.001	Fluoranthene
50	1-methylpyrene		0.001	Pyrene
51	benzo(c)phenanthrene		0.001	Phenanthrene
52	Benzo(ghi)fluoranthene		0.001	Fluoranthene
53	Cyclopenta(c,d)pyrene		0.001	Pyrene
54	Perylene		0.001	
55	3-methylcholanthrene		0.001	

Table S2. *Cont.*

PAH #	Compound	TEF	TEFs to isomers are assigned & literature	Used standard
56	Indeno[123-cd]fluoranthene		0.001	Fluoranthene
57	Picene		0.001	
58	Anthanthrene		0.001	
59	Triphenylene		0.001	
60	Coronene		0.001	
61	Anthracene	0.01	0.01	
62	2-methylanthracene		0.01	Anthracene
63	5-methylchrysene		0.01	Chrysene
64	9-phenylanthracene		0.01	Anthracene
65	6-methylchrysene		0.01	Chrysene
66	Chrysene	0.01	0.01	
67	3-methylchrysene		0.01	Chrysene
68	7-methylbenz(a)anthracene		0.01	Anthracene
69	7,12-dimethylbenz(a)anthracene		0.01	Anthracene
70	Benzo(b)chrysene		0.01	Chrysene
71	Benzo(ghi)perylene	0.01	0.01	
72	Benz(a)anthracene	0.1	0.1	
73	Benzo(b)fluoranthene	0.1	0.1	
74	Benzo(j)fluoranthene		0.1	OEHHA 1993
75	Benzo(k)fluoranthene	0.1	0.1	
76	Benzo(a)fluoranthene		0.1	Benzo(b)fluoranthene
77	Indeno[123-cd]pyrene	0.1	0.1	
78	Dibenzo(b,k)fluoranthene		0.1	Benzo(b)fluoranthene
79	BeP		1	BaP
80	BaP	1	1	
81	7-methylbenzo(a)pyrene		1	BaP
82	Dibenzo(ac)anthracene		1	Dibenzo(ah)anthracene
83	Dibenzo(ah)anthracene	1	1	
84	Dibenzo(a,j)anthracene		1	Dibenzo(ah)anthracene
85	Dibenzo(a,e)pyrene		1	OEHHA 1993
86	Dibenzo(a,l)pyrene		10	OEHHA 1993
87	Dibenzo(a,i)pyrene		10	OEHHA 1993
88	Dibenzo(a,h)pyrene		10	OEHHA 1993

Table S3. BaPeq calculated for samples collected for different projects.

	Project name												
	**A-1**	**A-2**	**B-1**	**T-1**	**T-2**	**M-1**	**E-1**	**E-2**	**E-3**	**E-4**	**E-5**	**Mi-1**	**Mi-2**
	**Number of samples**												
	44	6	11	46	50	7	71	16	26	17	9	16	6
**Statistical data**	**16 particle phase PAHs**												
**mean**	0.114	0.009	24.067	0.000	0.001	12.705	0.025	0.080	1.029	0.182	4.884	0.052	3.810
**std**	0.083	0.011	13.583	0.000	0.001	14.329	0.039	0.090	1.731	0.421	6.001	0.019	4.185
**min**	0.002	0.000	7.326	0.000	0.000	3.704	0.000	0.000	0.000	0.000	0.027	0.023	0.036
**25%**	0.040	0.000	14.705	0.000	0.000	4.767	0.001	0.001	0.004	0.001	0.037	0.043	0.087
**50%**	0.111	0.006	21.352	0.000	0.000	8.837	0.007	0.064	0.009	0.010	1.084	0.050	3.214
**75%**	0.154	0.014	31.166	0.001	0.001	11.276	0.035	0.126	0.472	0.210	11.441	0.056	7.056
**max**	0.373	0.028	54.678	0.001	0.004	44.307	0.240	0.308	4.660	1.741	13.355	0.100	9.012
	**16 gas phase PAHs**												
**mean**	0.17	0.44	5.38	0.00	0.00	2.90	4.60	0.14	3.10	0.72	49.21	3.69	5.83
**std**	0.10	0.63	1.87	0.00	0.00	6.07	8.17	0.09	5.91	1.00	52.55	1.48	3.59
**min**	0.00	0.15	1.65	0.00	0.00	0.04	0.07	0.02	0.01	0.06	3.21	1.38	1.62
**25%**	0.08	0.16	4.32	0.00	0.00	0.14	0.59	0.06	0.05	0.09	6.31	2.66	2.63
**50%**	0.16	0.19	5.67	0.00	0.00	0.84	1.36	0.13	0.09	0.20	9.49	3.53	6.41
**75%**	0.22	0.23	6.65	0.00	0.00	1.26	3.21	0.18	0.38	1.10	98.86	4.96	8.52
**max**	0.43	1.73	8.60	0.00	0.00	16.62	40.23	0.37	17.42	4.05	120.49	6.20	9.97
	**88 particle phase PAHs**												
**mean**	0.341	0.135	41.245	0.001	0.001	25.082	0.391	0.462	2.846	0.308	14.952	0.151	8.657
**std**	0.242	0.147	22.075	0.000	0.001	24.142	0.630	0.997	3.946	0.709	10.649	0.069	9.323
**min**	0.006	0.000	12.742	0.000	0.000	5.895	0.000	0.001	0.007	0.001	0.325	0.093	0.289
**25%**	0.125	0.015	26.921	0.000	0.000	11.698	0.009	0.010	0.035	0.003	4.440	0.107	0.339
**50%**	0.340	0.088	40.606	0.001	0.001	16.343	0.106	0.170	0.067	0.038	15.292	0.139	7.256
**75%**	0.500	0.266	49.564	0.001	0.002	27.642	0.568	0.292	6.608	0.347	26.176	0.159	16.016
**max**	1.083	0.316	93.431	0.002	0.007	74.656	2.918	3.947	9.805	2.933	28.777	0.363	20.171
	**88 gas phase PAHs**												
**mean**	0.385	0.685	15.464	0.001	0.001	5.329	17.096	0.355	11.629	1.109	194.113	7.753	14.283
**std**	0.250	0.639	8.368	0.001	0.001	10.963	15.102	0.187	21.395	1.337	218.190	4.576	7.285
**min**	0.005	0.286	2.736	0.000	0.000	0.114	1.718	0.138	0.014	0.108	10.912	2.063	5.233
**25%**	0.188	0.372	10.984	0.000	0.000	0.491	6.476	0.226	0.169	0.325	17.718	5.094	9.396
**50%**	0.350	0.477	15.300	0.001	0.001	1.504	11.968	0.302	0.263	0.538	19.642	6.792	13.469
**75%**	0.492	0.537	16.988	0.001	0.002	2.296	21.200	0.442	1.102	1.608	369.254	8.688	19.098
**max**	1.113	1.975	34.366	0.006	0.004	30.108	64.406	0.733	58.280	5.662	497.529	19.886	24.515

Table S4. Ratios of BaPeq calculated for samples collected for different projects.

	Project name												
	**A-1**	**A-2**	**B-1**	**T-1**	**T-2**	**M-1**	**E-1**	**E-2**	**E-3**	**E-4**	**E-5**	**Mi-1**	**Mi-2**
	**Number of samples**												
	**44**	**6**	**11**	**46**	**50**	**7**	**71**	**16**	**26**	**17**	**9**	**16**	**6**
**Statistical data**	**$BaPeq_{I}$: PM∑16BaPeq to PM∑88BaPeq**												
**mean**	0.346	0.299	0.575	0.556	0.465	0.514	0.210	0.260	0.227	0.478	0.210	0.375	0.327
**std**	0.079	0.394	0.049	0.037	0.091	0.159	0.260	0.176	0.180	0.187	0.214	0.134	0.138
**min**	0.067	0.008	0.489	0.477	0.044	0.235	0.000	0.052	0.012	0.032	0.006	0.124	0.124
**0.25**	0.317	0.039	0.550	0.526	0.446	0.447	0.026	0.076	0.062	0.424	0.011	0.303	0.244
**0.5**	0.368	0.110	0.585	0.561	0.479	0.593	0.091	0.295	0.175	0.520	0.084	0.373	0.351
**0.75**	0.398	0.440	0.603	0.587	0.510	0.622	0.313	0.393	0.414	0.583	0.398	0.437	0.445
**max**	0.464	1.000	0.650	0.617	0.644	0.629	1.000	0.541	0.498	0.775	0.508	0.728	0.448
	**$Gas\sum_{16}BaPeq$ to $Gas\sum_{88}BaPeq$**												
**mean**	0.303	0.012	1.722	1.458	0.314	24.032	0.002	0.265	0.414	0.075	0.024	0.009	0.521
**std**	0.169	0.013	0.668	2.899	0.291	40.666	0.002	0.315	0.754	0.090	0.031	0.009	0.679
**min**	0.034	0.000	0.621	0.106	0.021	0.294	0.000	0.001	0.002	0.001	0.002	0.001	0.001
**25%**	0.209	0.001	1.195	0.379	0.104	2.802	0.000	0.003	0.007	0.004	0.003	0.005	0.005
**50%**	0.278	0.009	1.942	0.591	0.153	9.432	0.001	0.165	0.070	0.055	0.016	0.007	0.348
**75%**	0.361	0.023	2.175	1.342	0.553	19.675	0.003	0.442	0.254	0.123	0.027	0.011	0.701
**max**	0.876	0.029	2.677	19.197	0.934	113.547	0.010	0.964	2.641	0.308	0.099	0.039	1.722
	**$BaPeq_{II}$: $PM\sum_{16}BaPeq$ to (PM+Gas)∑16BaPeq**												
**mean**	0.378	0.023	0.803	0.648	0.371	0.842	0.020	0.321	0.281	0.087	0.061	0.018	0.375
**std**	0.113	0.027	0.039	0.157	0.208	0.237	0.050	0.289	0.308	0.091	0.053	0.013	0.409
**min**	0.162	0.002	0.732	0.211	0.038	0.347	0.000	0.003	0.006	0.003	0.003	0.004	0.004
**25%**	0.300	0.003	0.774	0.565	0.203	0.811	0.001	0.009	0.025	0.011	0.006	0.009	0.010
**50%**	0.378	0.011	0.813	0.622	0.313	0.970	0.006	0.340	0.188	0.078	0.062	0.014	0.309
**75%**	0.426	0.042	0.832	0.744	0.583	0.977	0.018	0.556	0.366	0.139	0.102	0.021	0.750
**max**	0.647	0.063	0.864	0.995	0.675	0.997	0.307	0.773	0.914	0.301	0.142	0.054	0.823
	**$BaPeq_{III}$: $PM\sum_{16}BaPeq$ to $(PM+Gas)\sum_{88}BaPeq$**												
**mean**	0.148	0.008	0.419	0.308	0.156	0.413	0.002	0.103	0.074	0.058	0.016	0.009	0.151
**std**	0.051	0.008	0.074	0.105	0.099	0.160	0.002	0.114	0.100	0.063	0.016	0.008	0.165
**min**	0.032	0.000	0.285	0.088	0.020	0.199	0.000	0.001	0.002	0.001	0.001	0.001	0.001
**25%**	0.128	0.001	0.350	0.232	0.085	0.285	0.000	0.003	0.007	0.004	0.002	0.005	0.004
**50%**	0.145	0.008	0.459	0.295	0.119	0.465	0.001	0.070	0.057	0.052	0.015	0.007	0.137
**75%**	0.169	0.015	0.471	0.371	0.241	0.532	0.003	0.176	0.082	0.104	0.025	0.011	0.272
**max**	0.274	0.018	0.497	0.525	0.336	0.591	0.009	0.346	0.408	0.203	0.045	0.036	0.355

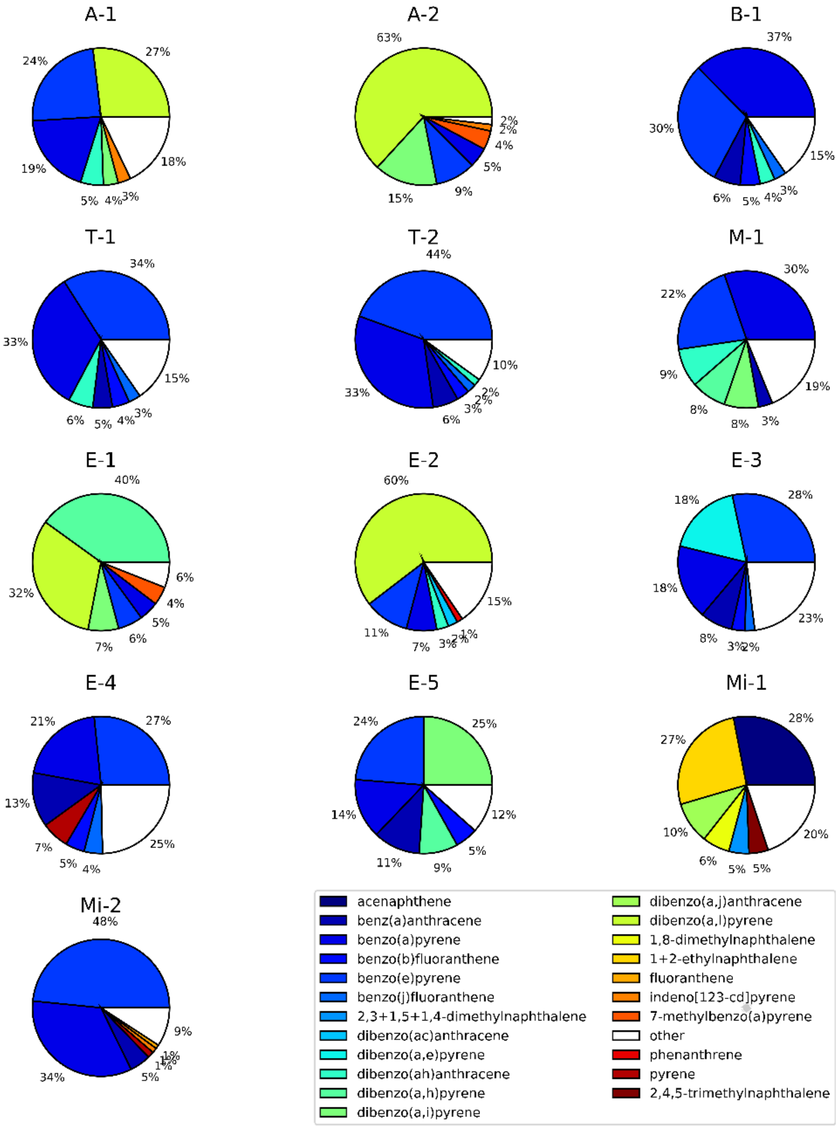

Figure S1. Top six PAHs that have highest BaPeq concentrations in particle-phase samples.

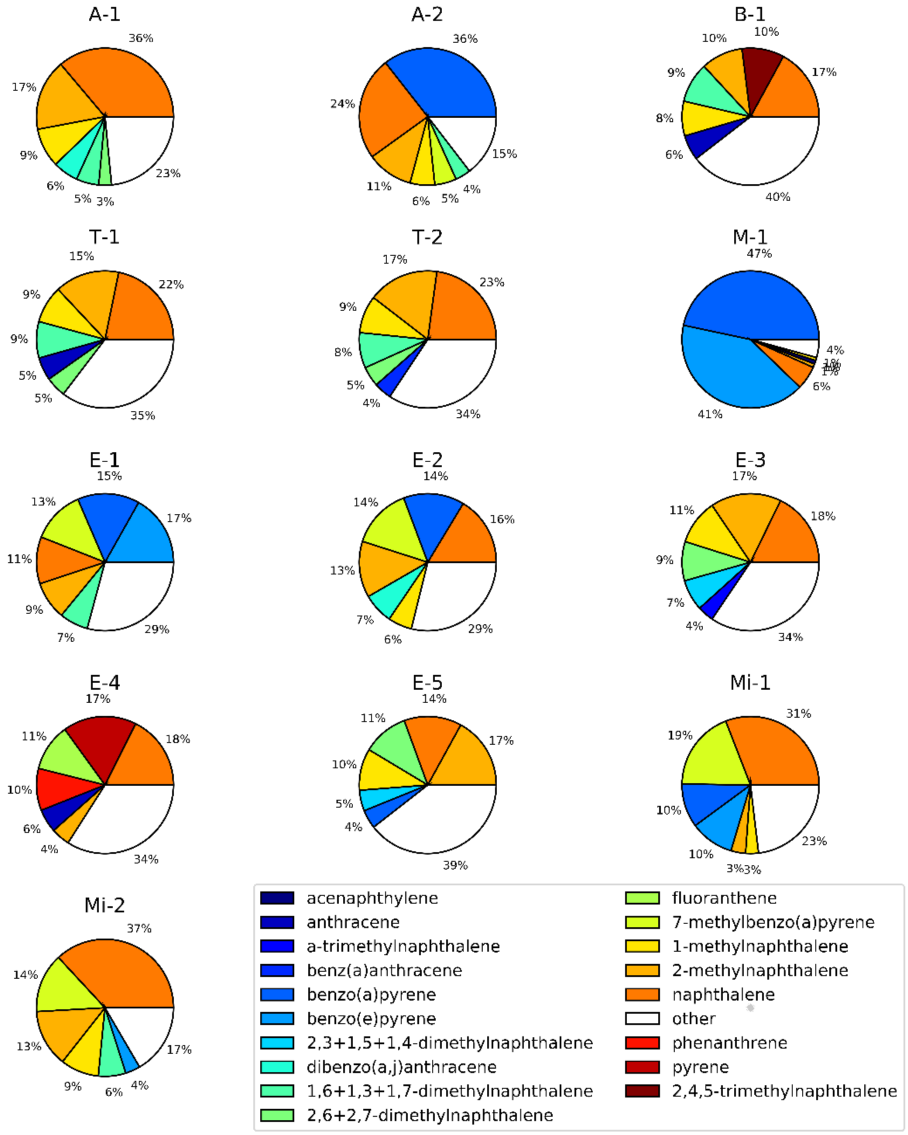

Figure S2. Top six PAHs that have highest BaPeq concentrations in gas-phase samples.
